# Oral microbiome diversity and composition before and after chemotherapy treatment in pediatric oncology patients

**DOI:** 10.1186/s12903-025-06405-4

**Published:** 2025-07-02

**Authors:** Egle Immonen, Lauri Paulamäki, Hannaleena Piippo, Atte Nikkilä, Liisa Aine, Timo Peltomäki, Olli Lohi, Mataleena Parikka

**Affiliations:** 1 Tampere Center for Child, Adolescent and Maternal Health Research, Faculty of Medicine and Health Technology, Tampere University, Tampere, Finland; 2 Faculty of Medicine and Health Technology, Tampere University, Tampere, Finland; 3 Department of Ear and Oral Diseases, Tampere University Hospital, Tampere, Finland; 4 Tays Cancer Center, Tampere University and Tampere University Hospital, Tampere, Finland

**Keywords:** Oral microbiome, Diversity, Chemotherapy, Randomized, Pediatric, Mucositis

## Abstract

**Objective:**

This study investigated the impact of anticancer treatment on the oral microbiome in pediatric patients and its association with oral mucositis (OM).

**Materials and methods:**

A double-blind, randomized trial involving 34 pediatric cancer patients (ages 2–17.99) with solid or hematological malignancies. Mucosal swab samples were collected before and after chemotherapy. Patients underwent two 7-day rinse cycles—one with Caphosol and one with saline—in a randomized order. Bacterial DNA from 110 mucosal swabs was analyzed using 16S rRNA sequencing.

**Results:**

Chemotherapy altered bacterial composition. No life-threatening OM cases (WHO grade 4) were observed, but mild to severe OM (grades 1–3) occurred in three patients. In patients without oral lesions, *Bergeyella* genus was more abundant prior to treatment while *Alloprevotella* was more abundant in the post-treatment samples, compared to patients with lesions. OM was linked to distinct microbiome profiles, including *Stenotrophomonas, Leptotrichia sp., Serratia sp.,Capnocytophaga sputigena, Sphingomonas sp., Parapusillimonas sp., Staphylococcus sp.*, and *Turicibacter* genera. Additionally, *Burkholderia-Caballeronia-Paraburkholderia* (*p* = 0.013) were more prevalent in the Caphosol group compared to the saline group.

**Conclusions:**

These findings indicate that chemotherapy-induced microbiome shifts associate with OM risk, highlighting the potential for microbial markers to predict high-risk patients and support protective strategies.

**Trial registration:**

The trial titled "Supersaturated Calcium Phosphate Oral Rinse (Caphosol®) for the Prevention of Oral Mucositis in Children Undergoing Chemotherapeutic Treatments" was registered on ClinicalTrials.gov (ID NCT02807337), with the first submission date 2016–06-07.

**Supplementary Information:**

The online version contains supplementary material available at 10.1186/s12903-025-06405-4.

## Introduction

The oral microbial community is the second most complex in the human body, surpassed only by the gut microbiome [1]. Through 16S rRNA sequencing and amplicon sequence variants (ASVs), over 700 species of oral microorganisms have been identified, classifying organisms based on DNA similarity [2, 3]. The Human Oral Microbiome Database (HOMD) provides comprehensive data on bacterial communities found in the oral cavity, pharynx, nasal passages, sinuses, and esophagus [4]. Oral microbial composition varies significantly between children and adults [5]. A substantial portion of the maturation of a child’s oral microbiome takes place during the first two years of life [6], and by the age of three, the salivary microbiome becomes more complex, continuing to evolve with age [7].

The oral microbiota is closely linked to both oral and systemic health. While microbial communities function similarly across individuals, they can exhibit considerable uniqueness and play different roles in healthy versus diseased states [8]. Analyzing the oral microbiota in different clinical conditions helps identify disease-associated microbial patterns, enabling patient-specific risk profiles and optimized personalized treatment strategies [9]. Pediatric cancer patients form a unique group where oral microbiota analysis offers key insights into prognosis and treatment-related complications like chemotherapy-induced oral mucositis (OM) [10, 11]. Over the past decade, research on the oral microbiome in pediatric cancer patients has grown substantially [12]. Nevertheless, OM remains one of the most common and debilitating complications of cancer treatment [13]. Indeed, recent studies have highlighted a potential causal relationship between the oral microbiome and mucositis in both pediatric and adult cancer patients [14–18]. For example, Oldenburg et al. [14] found that microbiome disruptions during treatment predict side effects, with Enterococcaceae dominance linked to infections. Laheij and Soet [15] associated oral ulcerative mucositis with periodontitis-related bacteria, particularly Porphyromonas gingivalis. Sonis [16] and Wand et al. [17] suggested that mucositis arises when commensal bacteria lose their protective role due to ecological imbalances in chemotherapy. While microbiome changes during cancer therapy are well-documented, their precise impact on mucositis progression remains unclear. These findings highlight the need for precise microbiome profiling and modulation to mitigate mucositis severity, reduce complications, and improve treatment efficacy.

In pediatric cancer patients, oral dysbiosis may arise due to antibiotic use, chemotherapy, and radiotherapy, affecting the mucosal barrier defenses [19, 20]. Changes to the oral microbiome may significantly influence susceptibility to infections in immunocompromised patients [21–23]. Some studies suggest that OM is not a direct consequence of chemotherapy or radiotherapy but rather a response to mucosal injury, which alters the local environment, making it more hostile to resident microbes [19, 24, 25]. The role of the oral microbiome in the development of mucositis in pediatric cancer patients remains incompletely understood. It is unclear whether chemotherapy-induced microbiome alterations contribute directly to mucositis pathogenesis or if they are a secondary consequence of treatment-related mucosal damage.

Various therapeutic strategies can be employed for both the prevention and management of OM, including oral hygiene protocols, antiseptic, antifungal, and anesthetic rinses or gels, cryotherapy, herbal treatments, anti-inflammatory agents, growth factors, cytokines, and photobiomodulation therapy (PBMT) [26]. Recently, Caphosol (Jazz Pharmaceuticals plc), a supersaturated calcium phosphate rinse, has gained popularity for its ability to penetrate the mucosa, reduce inflammation, and promote epithelial healing [27, 28].

There are no ideal methods for obtaining oral samples from children to ensure reliable results. However, various sample types, such as saliva and mucosal swabs, are commonly used to analyze the oral microbiome in pediatric patients. For infants and young children, these non-invasive methods are particularly suitable for diagnostic purposes [29–31].

The primary aim of our study was to analyze oral microbiome diversity and composition before and after chemotherapy in pediatric oncology patients using Caphosol or saline rinses. Mucosal swabs were collected to identify microbial features associated with OM.

## Materials and methods

### Subject selection and clinical outcomes

A total of 45 patients, aged between 2 and 17.99 years, with solid or hematological malignancies undergoing chemotherapy, participated in a multicenter, prospective, double-blind, randomized clinical trial (NCT02807337), which adhered to the Consolidated Standards of Reporting Trials (CONSORT) guidelines. The clinical outcomes of this trial have been previously reported [32]. The chemotherapy regimens included one of the following drugs: high-dose methotrexate (≥ 1 g/m^2^), any anthracycline (doxorubicin, daunorubicin, idarubicin, mitoxantrone), or cisplatin, along with at least two cycles of chemotherapy containing these agents, all of which are known to increase the risk of mucositis. All patients underwent two 7-day cycles of mouth rinses, administered four times daily—one with Caphosol and the other with a saline solution—in a randomized order. A minimum three-week interval between rinse cycles was maintained to ensure adequate mucosal healing between chemotherapy courses. Patients were excluded if they were presented OM at the start of chemotherapy, had undergone high-dose chemotherapy followed by stem cell transplantation (SCT), or were receiving induction chemotherapy for acute leukemia. OM was assessed using the World Health Organization (WHO) Oral Toxicity Scale, as described in previous studies [32–34]. Patient-reported outcomes were evaluated daily for two weeks (14 days) using the Children’s International Mucositis Evaluation Scale (ChIMES) [32, 35, 36]. ChiMES assesses OM in pediatric patients through 5 questions on pain, eating, drinking, and daily impact. Each question is answered using a 5-point Likert scale, ranging from no symptoms to severe symptoms, allowing children to self-report the severity of their condition.

### Sample collection and preparation

Mucosal swab samples were collected by a dentist, trained nurse, or pediatric oncologist before the start of chemotherapy and again at the end of the treatment course upon patient discharge. No exact time points were set for subsequent collections. Patients were allowed to eat and drink normally before sampling, with no restrictions on food, beverages, or oral hygiene practices prior to collection. At the time of sampling, all patients had already completed at least one cycle of chemotherapy.

**Fig. 1:**
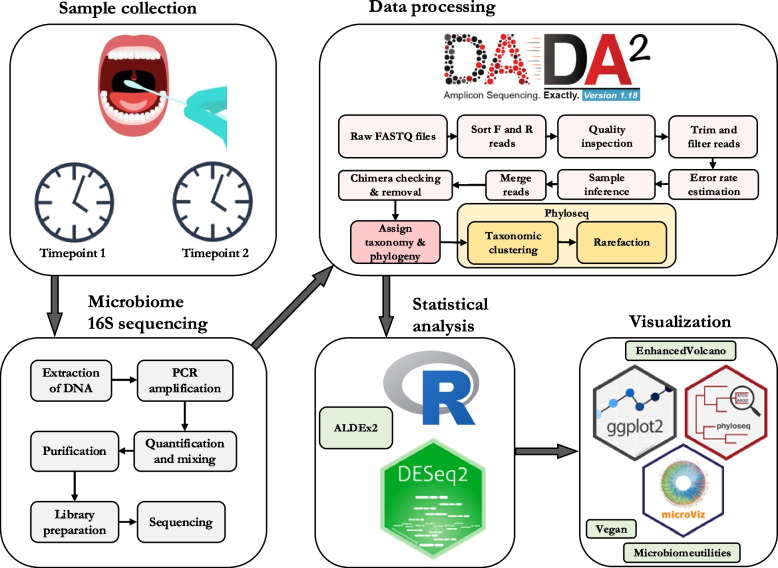
Workflow diagram illustrating the study process, divided into three main stages: sampling and laboratory work, data processing, and statistical analysis and visualization. Each stage is distinguished by a consistent color scheme for clarity. R packages utilized during data processing, analysis and visualization are written in subtitles with the version number of each package

This cross-over study involved collecting four mucosal swab samples from each patient, specifically from the mucosa beneath the tongue, using a cotton swab (M40 Transystem™ with Amies Agar Gel without charcoal) [37]. Samples were immediately frozen after collection and stored at -80 °C until further processing (Fig. 1). Informed consent was obtained from all patients, or from parents/legal guardians for young children, depending on the patient's age.

### DNA extraction for microbiome evaluation

Bacterial DNA was extracted from the cotton swab samples using the QIAamp DNA Mini Kit (Qiagen, California, USA) according to the manufacturer’s instructions. The total amount of bacterial DNA was determined by quantitative polymerase chain reaction (PCR) [38]. The extracted DNA was then stored at -20 °C prior to further analyses (Fig. 1).

### Next generation sequencing and data analysis

Bacterial DNA diversity was analyzed using Illumina NovaSeq next-generation sequencing with 250 bp paired-end chemistry, achieving a depth of 100 k reads per sample. Primers targeting the V3-V4 hypervariable regions of bacterial 16S rRNA (16S rDNA) were used for amplification (F341, 5ʹ-CCTAYGGGRBGCASCAG-3ʹ and R806, 5ʹ-GGACTACNNGGGTATCTAAT-3ʹ) [39]. All amplification steps, library preparation, sequencing, and data preprocessing were conducted by Novogene (Cambridge, UK). Before filtering stages, the mean number of raw reads per sample was 119,025 (sd 11,892) and after 99,607 (sd. 11,379), therefore 83.7% of the reads passed the quality, and chimera filtering steps (Figure S1).

Data processing and analysis of the reads was conducted in-house using the R programming language (4.3.1) with RStudio (2023.06.1 + 524) integrated development environment. Sequence quality and length-based filtering, chimera removal and taxonomy assignment were performed using the divisive amplicon denoising algorithm 2 (DADA2) package. (1.28.0) [40]. Filtering parameters in FilterAndTrim function were MaxN = 0, maxEE = c(2,2), truncQ = 2, rm.phix = TRUE, after the merging of read pairs, the sequences were filtered to only those that were within 403 and 431 bases in length. The reference taxonomy used was Silva No. 99 v138.1. Prior to downstream analysis, data was rarefactioned to sequencing depth of the smallest sample size 64,537, and the subsampling was performed 1000 times to calculate the average sequence table. Phyloseq (1.46.0) [41] was utilized to aggregate the ASV’s to the most probable phylogenies, to genus and phylum level. We used genus level aggregation for most downstream analysis, such as differential expression analysis based on a generalized linear model (DESeq2) (1.42.1) [42] that was employed for differential abundance analysis between rarefactioned and aggregated samples. To compare the effect of compositionality, analysis of compositions of microbiomes with bias correction (ALDEx2) (1.38.0.) package was utilized for non-rarefactioned data to perform centered logarithm ratio (CLR) transformations and downstream methods for analyzing the differential abundance between groups. The Benjamini–Hochberg method was applied for multiple test correction in all methods involving multiple testing and *p*-values presented in the results section is the corrected value.

Several R packages were used for data visualization, including microbiomeutilities (1.0.17) [43], which facilitated data formatting for subsequent visualization steps. Alpha diversity plots were created using microbiomeutilities and NMDS plot was drawn with the vegan package (2.6–6) [44]. Vegan was also used for beta-diversity metrics and statistical testing. EnhancedVolcano (1.20.0) [45], along with in-house R scripts, was used to visualize differential abundance analysis by DESeq2 in volcano plots. A combination bar plot of species-level abundances in the samples was generated using the MicroViz package (0.12.1) [46].

The English language was edited by using a large language model (chatGPT4.0 and Copilot).

## Results

### Characteristics of the study population

A total of 45 children and adolescents with a median age of 6.5 years (range 2.1–17.1 years; see Table 1) were randomized in the study [32]. Thirteen patients (29%) discontinued the study due to the mouth rinse’s unpleasant taste, severe nausea, vomiting, or overall weakness – six patients (13%) during the Caphosol cycle and seven patients (16%) during the saline solution cycle [32]. Thirty-two patients completed the study, each undergoing two full cycles of mouth rinsing. Additionally, two more patients were included, who only underwent one cycle of mouth rinsing, with two mucosal swab samples collected before and after anticancer treatment. In total, 130 mucosal swab samples were collected before and after treatment, and the analysis was performed on 110 samples. The analysis required that each patient have mucosal swab samples collected both before and after at least one treatment cycle. If a sample from either time point was missing, the corresponding paired sample was excluded from the analysis. Following this approach, each patient had either 0, 2, or 4 samples in the final analysis. In the study by Immonen et al. [32], no extremely severe, life-threatening cases of OM with a WHO score of 4 were observed. However, mild to severe OM (WHO scores 1–3) was reported in three patients, with oral symptoms (grade ≥ 3, ChiMES questions 1–4) were reported in 13% during the Caphosol cycle and 29% during the saline cycle. For further analysis, the mucosal swab samples were categorized into six groups (see Table 2).

**Table 1:** Baseline characteristics of the study cohort by rinse order allocation (participant’s age, sex, diagnosis, rinse cycle 1 and 2)

	TOTAL (*n* = 45)	Group 1 (*n* = 24)	Group 2 (*n* = 21)
		Caphosol → Saline	Saline → Caphosol
Age, Median(*range*)	6.5 (2.1, 17.1)	6.0 (2.1, 14.7)	7.2 (3.1, 17.1)
Sex, N (%)			
Female	20 (44%)	13 (54%)	7 (33%)
Male	25 (56%)	11 (46%)	14 (67%)
Diagnosis, N (%)			
Hematological malignancy	28 (62%)	13 (54%)	15 (71%)
Solid tumor	7 (16%)	4 (17%)	3 (14%)
CNS tumor	10 (22%)	7 (29%)	3 (14%)
Discontinued, N (%)	13 (29%)	6 (25%)	7 (33%)
Cycle 1			
Treatment, N (%)			
High-dose methotrexate	27 (60%)	13 (54%)	14 (67%)
Other chemotherapy	18 (40%)	11 (46%)	7 (33%)
Blood values, median (*IQR*)			
WBC (10^9^/l)	3.8 (2.8, 5.2)	3.5 (2.8, 4.6)	3.9 (3.3, 5.3)
ANC (10^9^/l)	1.6 (0.9, 2.3)	1.6 (0.9, 2.1)	1.5 (0.9, 2.3)
CRP (mg/l)	0 (0, 3)	0 (0, 1)	0 (0, 4.4)
Cycle 2			
Treatment, N (%)			
High-dose methotrexate	27 (60%)	14 (58%)	13 (62%)
Other chemotherapy	17 (38%)	10 (42%)	7 (33%)
Not available^a^	1 (2%)	0 (0%)	1 (5%)
Blood values, median (*IQR*)			
WBC (10^9^/l)	3 (1.9, 4.6)	2.5 (1.9, 4.3)	3.3 (2.3, 4.6)
ANC (10^9^/l)	1.2 (0.7, 2.1)	1.1 (0.8, 2)	1.4 (0.6, 2.1)
CRP (mg/l)	0 (0, 1.8)	0 (0, 1.4)	0 (0, 4.6)

CRP values coded as < 5 or < 1 were treated as zeros

*CNS* Central nervous system, *IQR* Interquartile range, *WBC* White blood cell count, *ANC* Absolute neutrophil count, *CRP* C-reactive protein

^a^Subject had finished his treatment protocol before the second chemotherapy cycle

**Table 2:** Group classification of oral samples based on mucosal lesions development and mouth rinse treatment

Group Description	Inclusion Criteria
Pretreatment Samples – Patients with Oral Mucosal Lesions	WHO score ≥ 1 and ChIMES grade ≥ 1
Pretreatment Samples – Patients without Oral Mucosal Lesions	WHO score = 0 and ChIMES grade = 0
Posttreatment Samples – Patients with Oral Mucosal Lesions	WHO score ≥ 1 and ChIMES grade ≥ 1
Posttreatment Samples – Patients without Oral Mucosal Lesions	WHO score = 0 and ChIMES grade = 0
Posttreatment Samples – Patients using Caphosol Mouth Rinse	Treatment group using Caphosol
Posttreatment Samples – Patients using Saline Solution	Treatment group using saline solution (NaCl)

### Alpha-diversity and richness of oral microbiota in pediatric cancer patients

Alpha diversity is essential in microbial analysis because it shows the richness and distribution of species within a community, helping to understand its complexity and health implications. It can indicate how treatments, such as chemotherapy, affect microbial communities. A reduction in alpha diversity may signal a loss of beneficial microbial species, potentially leading to conditions like mucositis [47].

**Fig. 2:**
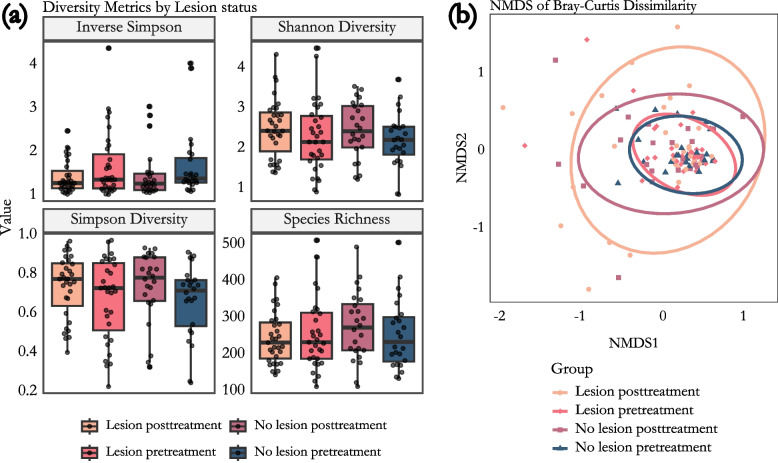
Diversity plots for the oral microbiota of pediatric cancer patients are presented. **a** Plots of inverse Simpson, Shannon diversity, Simpson diversity, and species richness are shown for all patients included in the study, with samples categorized by pre-treatment and post-treatment phases and further divided based on mucosal status (no lesions vs. lesions). No statistically significant differences were observed among any of the groups. **b** The NMDS1 plot illustrates samples grouped by mucosal status and sampling time (pre-treatment vs. post-treatment), revealing no statistically significant clusters

We analyzed the alpha diversity of mucosal swab samples collected from patients with and without oral lesions, both before and after anticancer treatment. The alpha diversity analysis revealed statistically significant differences in sampling time (Shannon; *p* = 0,079 & *p* = 0.29, Simpson; *p* = 0,040 & *p* = 0.0) however, neither lesion status nor oral rinse influenced alpha diversity statistics (Shannon; *p* = 0,69 & *p* = 0.29, Simpson; *p* = 0,90 & *p* = 0.087, respectively) (Fig. 2A, Table S4). Additionally, the consistently similar alpha diversity values suggest that chemotherapy did not lead to significant changes at the overall microbiome level (Fig. 2A). Non-metric multidimensional scaling (NMDS) analysis showed no clear clustering (Fig. 2B). However, PERMANOVA analysis revealed that while some of the variance is explained by sampling time (Bray–Curtis; *R*^*2*^ = 2.94%, *p* = 0.001, Weighted UniFrac; *R*^*2*^ = 4,21%, *p* = 0.005, betadispersion; *p* = 0.00084), most of the variance were attributed to paired nature of data (Bray–Curtis; *R*^*2*^ = 61,24%, *p* = 0.001, Weighted UniFrac; *R*^*2*^ = 59,34%, *p* = 0.002). Although there was a slight increase in deviation between the groups with and without lesions after treatment, the alpha diversity of the oral microbiota did not differ significantly between patients with lesions and those without (*p* = 0.56), nor between the pre-treatment and post-treatment time points (*p* = 0.69) (Fig. 2B).

### Abundance differences at phylum level in oral microbiota of patients with and without mucosal lesions

**Fig. 3:**
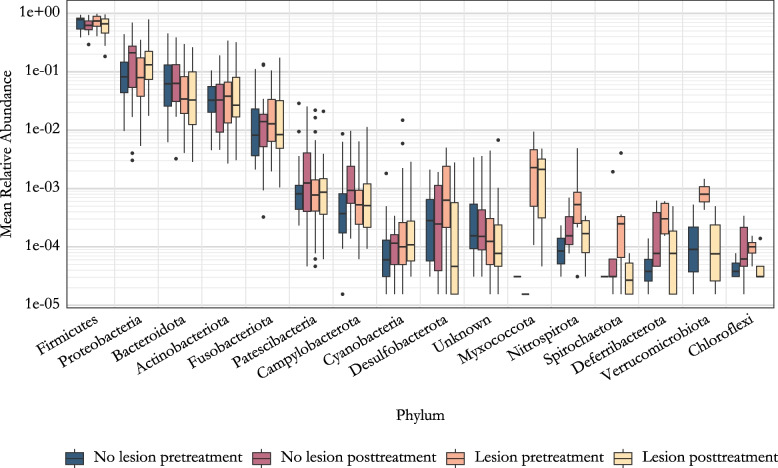
Mean relative abundance of phyla. The data is grouped by the lesion status and the sampling time. Phyla are ordered in decreasing order based on total read count

Next, we conducted a comparison of the oral microbiota between pediatric cancer patients with and without oral lesions, both before and after anticancer treatment, across different taxonomical levels (Fig. 3, Table S1). In the pre-treatment samples, we identified significant differences in the relative abundance of one bacterial phylum, with Bacteroidota being more abundant in the group without lesions (*p* = 0.034). In the post-treatment samples, no significant differences were observed between the patients with and without lesions. However, our study population exhibited a statistically significant increase in the relative abundance of the Proteobacteria phylum during cancer treatment among all participants, including those with and without oral lesions (*p* = 0.0023). Our study design allowed for pairwise testing, and thus we also tested differential abundance between sampling groups, lesion term and oral rinse, utilizing this feature of the data. However, none of the phyla reached statistical significance in those comparisons (Table S1).

### Abundance differences at genus-level in oral microbiota of patients with and without mucosal lesions

**Fig. 4:**
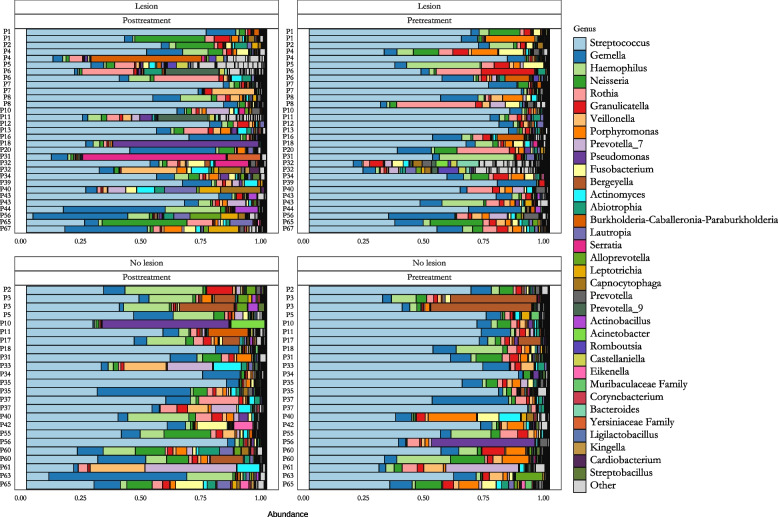
The microbial composition of individual samples is represented in the figure as a proportion of the total reads observed. Samples from patients without oral lesions are displayed at the top, while those with observed oral lesions are positioned at the bottom. Samples collected prior to the treatment regimen are shown on the left, and those collected post-treatment are on the right. Each row corresponds to a single sample, identified by patient ID. The 35 most abundant genus-level taxa are highlighted in color. Adjusted *p*-values from the differential abundance analysis are presented in a table, where each column corresponds to a specific pairwise comparison

**Fig. 5:**
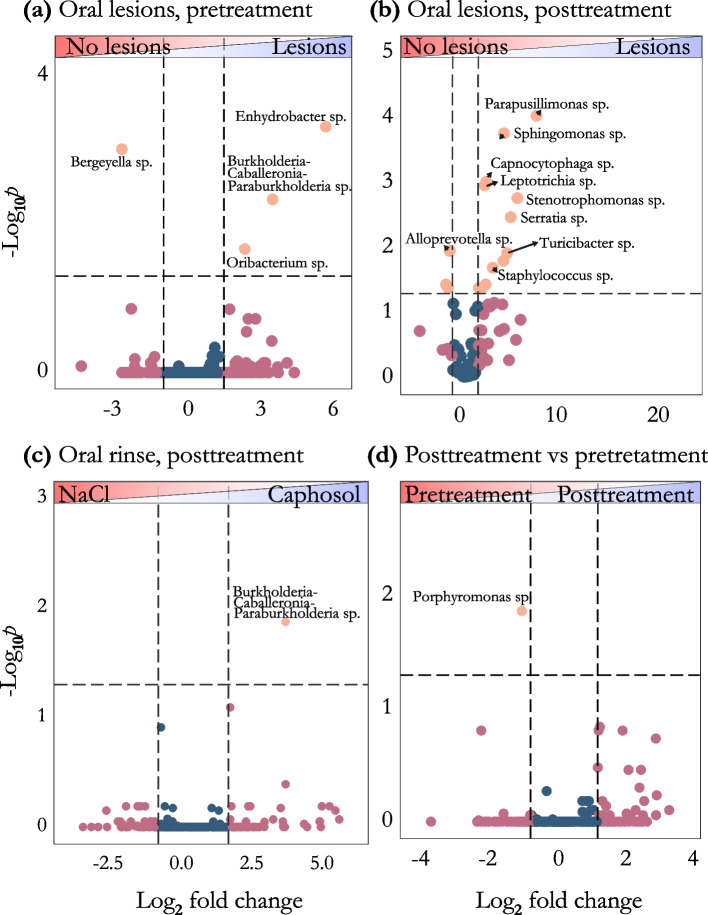
In this panel, taxa are illustrated in a volcano plot, which compares two groups of samples. The x-axis represents the fold change, while the y-axis displays significance as the negative logarithm to the base 10 of the *p*-value. **a** Depicts the differences in taxon abundance between pre-treatment samples with lesions and those without. **b** Shows the lesion status comparison in post-treatment samples (**c**) Illustrates the differences in taxon abundance between the saline solution and Caphosol oral rinse groups within post-treatment samples (**d**) Compares pre-treatment and post-treatment samples

The composition of the oral microbiome typically varies greatly between individuals. Figure 4 presents the 35 most abundant genus-level taxa from the samples included in the analysis. Visualization highlights the significant variability in microbiome composition between patients, as well as the changes occurring in the microbiomes of individual patients during treatment. In the pre-treatment samples, we observed statistically significant differences in the relative abundance of four bacterial genera when comparing patients with oral lesions to those without (Figs. 4 and 5A, Table S2). Differences were observed in the abundance of* Bergeyella sp*. (*p* = 0.00097), *Enhydrobacter sp.* (*p* = 0.00048), *Burkholderia-caballeronia-paraburkholderia* sp. (*p* = 0.0046), *Oribacterium* (*p* = 0.021). Interestingly, the abundance of *Bergeyella* was higher in the pre-treatment samples from patients without oral lesions (*p* = 0.00097) (Fig. 5A). Overall, these results indicate that the development of oral mucositis during anticancer treatment is associated with distinct oral microbiota profiles present before treatment.

We also investigated the changes in the abundance of oral microbiota following anticancer treatment. Our comparison of genus-level taxa revealed statistically significant differences in the abundance of eleven taxa (Figs. 4 and 5B, Table S2). Several taxa were found to be more abundant in patients with oral lesions, including *Stenotrophomonas* (*p* = 0.0023), *Leptotrichia sp*. (*p* = 0.013), *Serratia sp.* (*p* = 0.0068), *Capnocytophaga sp.* (*p* = 0.012), *Sphingomonas sp*. (*p* = 0.0024), *Parapusillimonas sp.* (*p* = 0.00013), *Staphylococcus sp.* (*p* = 0.028), and *Turicibacter sp.* (*p* = 0.021) (Fig. 5B). Conversely, in patients without lesions, the relative abundance of *Alloprevotella sp*. (*p* = 0.014) was found to be higher compared to patients with oral lesions (Fig. 5B). This genus is part of the protective normal microbiome of the oral cavity in healthy children [48, 49].

Moreover, all patients in this study, regardless of whether they developed oral lesions, exhibited a statistically significant decrease in the relative abundance of *Porphyromonas* (*p* = 0.014) during anticancer treatment (Fig. 5D, Table S2).

Considering the paired nature of the samples, the comparison of post- and pre-treatment samples revealed that some higher abundance taxa, such as *Streptococcus sp*. and *Granulicatella sp*., showed a slight decrease during treatment—an effect that might not have been detectable without the paired design (Table S2). Additionally, the paired design highlighted some low abundance taxa, whose relevance to oral health remains questionable.

### Abundance differences of oral microbiota between the Caphosol and saline solution oral rinse groups

From the post-treatment samples, we analyzed the differences in abundance of genus-level taxa between the Caphosol and saline solution groups. The abundance of *Burkholderia-Caballeronia-Paraburkholderia *(*p* = 0.013) was significantly higher in the Caphosol group compared to the saline solution group (Fig. 5C).

We also calculated all *p*-values using centered log-ratio transformations. No statistically significant differences were observed in the comparisons between any of the sample groups (Table S3).

## Discussion

In this follow-up to a previously published randomized, controlled, double-blind trial, we analysed 110 mucosal swab samples to evaluate the impact of chemotherapy on the oral microbiota. Our findings reveal that paediatric cancer treatment alters the oral microbiome at both the genus and phylum levels, leading to notable changes in its composition and diversity. Specifically, we found that the development of oral mucositis during anticancer treatment was associated with distinct oral microbiota profiles present before treatment, as well as shifts in the microbiota following treatment. These observations suggest that dysbiosis, both pre- and post-treatment, may play a critical role in the development of oral lesions.

Previous research has reported reductions in microbial diversity and compositional shifts in several oral taxa during anticancer treatment [14, 16, 17]. Dysbiosis disrupts the homeostasis of the oral epithelial barrier, impairing its response and accelerating pathological processes. Studies suggest that specific bacterial changes may contribute to OM development and delay ulcer healing [50, 51]. However, our study focuses on microbiome composition changes in response to chemotherapy and rinse types, rather than the biological mechanisms behind these effects. Despite OM’s significant clinical impact, its underlying mechanisms remain poorly understood.

In this study, no significant differences in alpha diversity were observed in oral samples collected before and after anticancer treatment in patients with mucosal lesions. Likewise, the richness of the oral microbiota remained stable in pediatric cancer patients, likely because participants may have already undergone microbiome alterations due to prior chemotherapy [52, 53]. Additionally, cancer itself may have affected microbiome diversity, which could account for the absence of significant differences in alpha diversity [54]. Findings from Laheij et al. [55], Wang et al. [52], Ye et al. [22], and de Farias Gabriel et al. [18] partly support our results but also highlight context-specific variations. Laheij et al. [55] reported a marked reduction in alpha diversity one-week post-SCT, whereas our study did not observe significant changes before and after treatment. This discrepancy may be due to differences in patient populations, treatments, and sampling time points. However, their findings that reduced alpha diversity correlated with shifts in key bacterial genera align with the idea that microbiome composition, rather than diversity alone, plays a role in treatment outcomes. Wang et al. [52] found dysbiosis with reduced richness and diversity in pediatric ALL patients compared to healthy controls, suggesting that pre-existing microbiome alterations due to cancer itself may explain the lack of alpha diversity changes in our study. In contrast, Ye et al. [22] showed that higher microbial diversity at diagnosis was associated with later mucositis development, with significant microbiome shifts post-chemotherapy before mucositis onset. While we did not observe similar microbial richness changes, their study reinforces the idea that baseline microbiome differences may influence mucositis risk, supporting our hypothesis that patients may have already undergone microbiome alterations before sampling. Meta-analyses such as de Farias Gabriel et al. [18] further illustrate the complex relationship between chemotherapy, microbiome alterations, and OM in pediatric oncology patients. Their findings emphasize microbial dysbiosis as a key factor in mucositis pathogenesis while also noting that differences in methodologies, sample collection, and treatment regimens contribute to inconsistencies in observed alpha diversity changes.

Another important finding from our study was the lower abundance of *Bergeyella* in patients who developed oral lesions compared to those who did not. *Bergeyella*, a Gram-negative, rod-shaped bacterium, is a key component of the oral microbiota [56]. Bruno et al. [57] suggested that *Bergeyella* could be a potential target for oral mucositis prevention. Our results, combined with their findings, suggest that *Bergeyella* may have a protective role against oral mucositis and microbiome dysbiosis, though further research is needed to confirm this.

Additionally, we observed an increased abundance of *Stenotrophomonas*, *Leptotrichia sp*., *Serratia sp.*, *Capnocytophaga sputigena*, *Sphingomonas sp*., *Parapusillimonas sp.*, *Staphylococcus sp.*, and *Turicibacter sp* in patients who developed oral lesions during anticancer treatment. The genus *Stenotrophomonas* comprises multidrug-resistant opportunistic pathogens found in aquatic environments, often contaminating hospital equipment. These bacteria are aerobic, non-fermentative, and Gram-negative [58]. Prates et al. [59] present a case of oral cavity infection caused by *S. maltophilia i*n an immunosuppressed patient. *Capnocytophaga* and *Leptotrichia* taxa are Gram-negative bacteria that have been linked to oral mucositis in children with neutropenia or malignancy [15, 22, 60]. *Leptotrichia* species, typically found in the oral cavity, have been linked to mucositis and other pathologies, particularly in immunocompromised individuals [61].

Conversely, we observed significant changes in the composition of oral microbiota among patients undergoing anticancer treatment, affecting both those with and without oral lesions. Notably, the abundance of *Alloprevotella sp.* increased during treatment in patients without lesions. While *Alloprevotella rava,* which has been identified in patients with oral dysbiosis, may contribute to these changes, *Alloprevotella* is generally considered a normal inhabitant of the oral cavity. Its precise role, however, remains unclear [14, 62].

Next, we observed a significant reduction in the relative abundance of *Porphyromonas* taxa among all patients in this study, regardless of whether they exhibited oral lesions during anticancer treatment. *Porphyromonas* taxa are Gram-negative oral pathogens associated with periodontitis and various systemic diseases [63, 64]. Notably, Bruno et al. [50] found a positive correlation between *Porphyromonas* and ulcerative oral mucositis. The decline observed in our study may be attributed to chemotherapy-induced disruptions in oral microbial diversity, resulting in an overall decrease in microbial abundance, including *Porphyromonas* species [65]. Furthermore, the immunocompromised status of pediatric cancer patients may lead to alterations in bacterial populations, further shifting the oral microbiota in response to treatment-related immune changes [12].

Moreover, we observed that the relative abundance of *Burkholderia-Caballeronia-Paraburkholderia* was higher in the Caphosol group compared to the saline solution group. *Burkholderia, Caballeronia, and Paraburkholderia* are genera of bacteria that can be found in various environments, including the oral cavity [66]. Certain species within the *Burkholderia* genus, particularly *Burkholderia cepacia*, are recognized as opportunistic pathogens that predominantly affect immunocompromised patients and have been identified in oral biofilms [67, 68]. The precise role of these organisms in oral health is not yet fully elucidated, though they may be implicated in dysbiotic states [66]. *Caballeronia* and *Paraburkholderia* have received limited attention in oral microbiome research, yet they may play a role in shaping the complex microbial communities associated with oral biofilms [69].

Our study has several limitations. The most notable is the small sample size, which may have affected the precision of our estimates. Additionally, all participants had undergone at least one cycle of chemotherapy before their initial oral sample was collected, and a healthy control group was not included for comparison.

Our results indicate that a higher risk of OM is associated with an increased relative abundance of *Bergeyella* prior to anticancer treatment and *Alloprevotella* post-treatment in mucosal swab samples collected from patients without oral lesions. Additionally, there was an increased abundance of *Capnocytophaga* and *Leptotrichia* taxa after treatment. These findings indicate that anticancer therapy, alongside a dysbiotic oral microbiome, may significantly influence the incidence, severity, and management of mucositis. Identifying patients at high risk for OM could enable improved treatment strategies by fostering protective bacterial species and reducing harmful ones. Further research is needed to clarify the microbiome characteristics that heighten the risk of OM, particularly in paediatric cancer patients.

## Supplementary Information

Supplementary Material 1.

Supplementary Material 2.

Supplementary Material 3.

Supplementary Material 4.

Supplementary Material 5.

Supplementary Material 6.

## Data Availability

All data generated or analyzed during this study are included in this article. A list of papers obtained from the electronic search, as well as the list of excluded studies and the reasons for their exclusion, are available from the authors upon request. Contact details: Egle Immonen, Tampere University, Tampere, Finland; email: egle.immonen@tuni.fi.

## Abbreviations

ALDEx2: Analysis of compositions of microbiomes with bias correction
ALL: Acute lymphoblastic leukemia
ASVs: Amplicon sequence variants
DADA2: Divisive amplicon denoising algorithm 2
DESeq2: Differential expression analysis based on a generalized linear model
CLR: Centered logarithm ratio
CONSORT: Consolidated Standards of Reporting Trials
CVCs: Central venous catheters
DNA: Deoxyribonucleic acid
HSCT: Hematopoietic stem cell transplantation
NGS: Next generation sequencing method
PCR: Polymerase chain reaction
rDNA: Recombinant deoxyribonucleic acid
rRNA: Ribosomal ribonucleic acid
RAS: Recurrent aphthous stomatitis
VGS: Viridans group streptococci
WHO: World Health Organization
