# Supplementary figures and images for: Oral microbiome diversity and composition before and after chemotherapy treatment in pediatric oncology patients
Source: BMC Oral Health. 2025 Jul 2;25:981. doi: 10.1186/s12903-025-06405-4 (PMC12220142; doi:10.1186/s12903-025-06405-4)

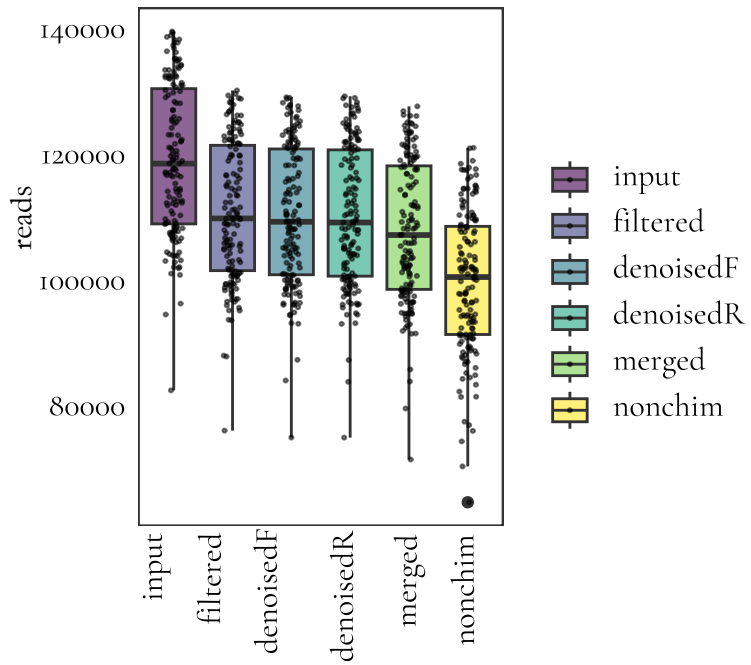

Supplement: Supplementary file 2 — Supplementary Material 2. [file 12903_2025_6405_MOESM2_ESM.pdf]
